# Analysis of B Cell Repertoire Dynamics Following Hepatitis B Vaccination in Humans, and Enrichment of Vaccine-specific Antibody Sequences

**DOI:** 10.1016/j.ebiom.2015.11.034

**Published:** 2015-11-24

**Authors:** Jacob D. Galson, Johannes Trück, Anna Fowler, Elizabeth A. Clutterbuck, Márton Münz, Vincenzo Cerundolo, Claudia Reinhard, Robbert van der Most, Andrew J. Pollard, Gerton Lunter, Dominic F. Kelly

**Affiliations:** a Oxford Vaccine Group, Department of Paediatrics, University of Oxford and the NIHR Oxford Biomedical Research Center, Oxford OX3 7LE, United Kingdom; b Welcome Trust Centre for Human Genetics, University of Oxford, Oxford, OX3 7BN, United Kingdom; c Paediatric Immunology, University Children's Hospital, Zürich, 8032, Switzerland; d Medical Research Council Human Immunology Unit, Weatherall Institute of Molecular Medicine, Oxford OX3 9DS, United Kingdom; e Miltenyi Biotec, Bergisch Gladbach, Germany; f GSK Vaccines, Rixensart, Belgium

**Keywords:** Vaccination, Immunoglobulin repertoire, B cell repertoire, mAbs

## Abstract

Generating a diverse B cell immunoglobulin repertoire is essential for protection against infection. The repertoire in humans can now be comprehensively measured by high-throughput sequencing. Using hepatitis B vaccination as a model, we determined how the total immunoglobulin sequence repertoire changes following antigen exposure in humans, and compared this to sequences from vaccine-specific sorted cells. Clonal sequence expansions were seen 7 days after vaccination, which correlated with vaccine-specific plasma cell numbers. These expansions caused an increase in mutation, and a decrease in diversity and complementarity-determining region 3 sequence length in the repertoire. We also saw an increase in sequence convergence between participants 14 and 21 days after vaccination, coinciding with an increase of vaccine-specific memory cells. These features allowed development of a model for in silico enrichment of vaccine-specific sequences from the total repertoire. Identifying antigen-specific sequences from total repertoire data could aid our understanding B cell driven immunity, and be used for disease diagnostics and vaccine evaluation.

## Highlights

• There is B cell immunoglobulin sequence expansion and mutation 7 days after vaccination.
• There is sequence convergence between participants 14 and 21 days after vaccination with a common antigen.
• These properties allow de novo identification of vaccine-specific immunoglobulin sequence clusters.

The B cell repertoire is a diverse system that adapts in response to infection/vaccination to protect against disease. Next-generation sequencing can be used to study this system at the level of the B cell immunoglobulin sequences, but it is difficult to distinguish the sequences responding to the antigen of interest from background noise. By sequencing both the total and vaccine-specific B cell immunoglobulin repertoire, we show that there are time-limited perturbations in the total repertoire following vaccination. These data were used to develop models for the enrichment of vaccine-specific sequences, which could give insight into the underlying biology of vaccination.

## 1 Introduction

The human B cell immunoglobulin repertoire has the theoretical potential to include up to 10^11^ unique variants (Glanville et al., 2009); such diversity is key to confer immunity against the variety of antigens that may be encountered during a lifetime. Upon antigen recognition, cognate B cells become activated, proliferate, and differentiate to produce short-lived antibody-secreting plasma cells (PCs) important in the initial response, and long-lived PCs and memory cells that contribute to sustained immunity. The use of vaccines as a controlled model system to study B cell responses is an important approach in human immunology (Lambert et al., 2005). B cell responses to vaccination are conventionally assessed by global measures of the quantity and function of specific antibody in the blood, but these provide little insight into the characteristics of the B cells responsible for producing the antibody, or their kinetics over time. Understanding which B cells produce specific antibody, and their kinetics in response to antigen encounter has application in the field of vaccine development and evaluation, and also in assessing response to infection (Galson et al., 2014).

Antibody specificity is largely determined by the immunoglobulin heavy chain gene sequence used by each individual B cell (Xu and Davis, 2000). Recent improvements in throughput, and decreases in cost of next-generation sequencing make it feasible to use this technology to characterize the immunoglobulin heavy chain repertoire in large numbers of samples. This approach has already yielded clinical applications in the monitoring of minimal disease residue in B cell lymphoma patients (Boyd et al., 2009), and the rapid identification of monoclonal antibody sequences (Reddy et al., 2010), and has shown promise for increasing understanding of autoimmune conditions (Palanichamy et al., 2014) and in disease diagnostics (Parameswaran et al., 2013).

High-throughput immunoglobulin sequencing is now also being applied to vaccine studies (Ademokun et al., 2011, Jiang et al., 2013, Laserson et al., 2014, Trück et al., 2015, Vollmers et al., 2013, Wang et al., 2014), and it appears possible to detect vaccine-induced perturbations in the total repertoire that relate to the functional B cell response (Jackson et al., 2014, Lavinder et al., 2014). Of interest, despite immunoglobulin repertoire diversity, there appears to be a degree of sequence convergence across individuals for a given antigen. Convergence has been seen seven days following vaccination with simple polysaccharide antigens (Trück et al., 2015), and more complex influenza antigens (Jackson et al., 2014), as well as following dengue infection (Parameswaran et al., 2013). It remains unclear how long perturbations in the immunoglobulin repertoire can be detected following vaccination, and whether such perturbations in the global repertoire can be used to identify the specific immunoglobulin sequences of the B cells generating the response. The ability to de novo enrich for immunoglobulin sequences with certain specificities from the total repertoire is key for understanding specific B cell responses to vaccination, and uncovering the clinical potential of this technology.

**Fig. 1:**
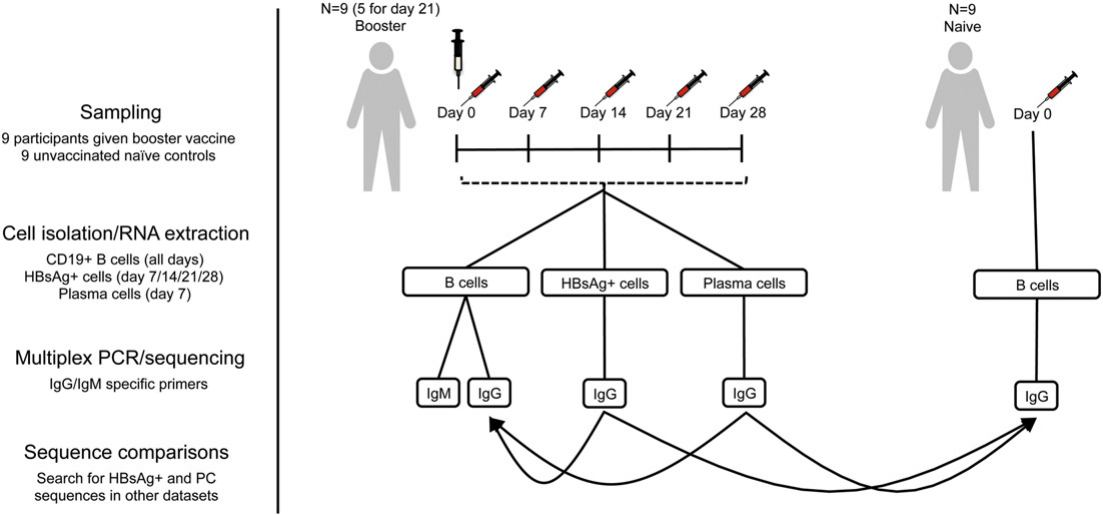
Study design. Two groups of participants were enrolled into the study: one group of nine participants with no previous HepB vaccine history (naïve group), and one group of nine participants who had previously received a course of HepB vaccine (booster vaccine group). In the naïve group, total B cells were sorted and sequenced (IgG transcripts only) at a single timepoint. In the booster vaccine group, total B cells were sorted and sequenced (IgG and IgM transcripts) on the day of HepB booster vaccine, and on days 7, 14, 21 (samples missing from 4 participants), and 28 following vaccination. In addition, for 5 of the participants in this group, HBsAg + B cells were isolated and sequenced on days 7, 14, 21 and 28 following vaccination, and PCs on day 7 following vaccination. Sequences from the HBsAg + sorted cells, and sorted PCs were used to find sequence clusters in the total repertoire of both the vaccinated and naïve group that appeared to have enriched specificity towards the vaccine.

We used the response to hepatitis B (HepB) vaccination as a model system to interrogate the global immunoglobulin heavy chain repertoire at multiple time points up to one month following booster vaccination with monovalent HepB surface antigen (HBsAg; Fig. 1). We show that the repertoire is variable between individuals, but that there are distinct signatures seen seven days following vaccination that appear to relate to the increase in the number of HBsAg-specific PCs in the peripheral blood at this time. We confirm that there is an increase in repertoire convergence present from at least day 14 until day 21 days following vaccination, more in keeping with the kinetics of HBsAg-specific memory cells in the peripheral blood. In addition to analyzing sequences from total B cells, we performed cell sorting of HBsAg-specific B cells and PCs, and sequenced these to investigate how their repertoire characteristics related to the perturbations seen in the samples derived from unsorted B cells. Furthermore, the B cell repertoire of HepB vaccine naïve participants was also sequenced, and differences were found in this population compared to the previously vaccinated population even at baseline. Using this information we were able to develop a simple analytic model to enrich for HBsAg-specific sequences from the repertoire data obtained from total B cells.

## 2 Materials & Methods

### 2.1 Sample Collection

Two groups with nine healthy subjects (aged 20–59) in each were recruited with informed consent, under approval from the Northampton Research Ethics Committee (13/EM/0036). One group consisted of participants with no prior HepB vaccine history (naïve group), who had blood taken at a single timepoint (Fig. 1). The second group consisted of participants who had previously received a primary course of HepB vaccination (vaccine group). Participants in this group were given a single intramuscular HepB booster vaccine containing 10 μg HBsAg, adsorbed on amorphous aluminum hydroxyphosphate sulfate (HBvaxPRO®, Sanofi Pasteur). Blood was taken immediately before vaccination as well as 7, 14, 21 and 28 days after vaccination. Blood was transferred to a heparinized tube for processing within 4 h of collection.

### 2.2 Anti-HBs Testing

Blood serum was isolated by centrifugation, and tested for anti-HBs IgG concentration by ELISA at the microbiology laboratory, John Radcliffe Hospital, Oxford, UK.

### 2.3 Enumeration of Plasma and Memory Cells by ELISpot

Peripheral blood mononuclear cells (PBMCs) were isolated from blood by density-gradient centrifugation over lymphoprep (Asis-Shield Diagnostics). Cultured and ex-vivo ELISpot assays were conducted from the PBMCs to detect antigen-specific memory and PCs respectively, using a previously described protocol (Clutterbuck et al., 2012). Briefly, MultiScreen-IP 96-well plates (Millipore) were coated with 2.5 μg/ml of purified HBsAg (GSK). For enumeration of HBsAg-specific PCs, 200,000 PBMCs were placed in each well, and incubated overnight. Cells were then washed from the plate, and IgG antibody bound to the plate detected using the AP conjugate substrate kit (Bio-Rad). For enumeration of HBsAg-specific memory cells, PBMCs were first cultured in RPMI (Sigma-Aldrich) containing 1/10 NBBS, 1/5000 *Staphylococcus aureus* cowans strain, 1/6000 pokeweed mitogen (Sigma-Aldrich) and 1/40 CpG class B oligonucleotide (5′-tcgtcgttttgtcgttttgtcgtt-3′; InvivoGen) for 6 days to activate antibody secretion. 200,000 cultured cells were added to each well of the plate, and detected in the same way as the ex-vivo cells. Spot counting was performed automatically using the AID ELISpot Reader System (AID Autoimmun Diagnostika). For each sample, 6 wells were seeded in parallel, and the mean spot count taken.

### 2.4 Cell Sorting

B cells were enriched from PBMCs using CD19 microbeads (Miltenyi Biotec), and the AutoMACS Pro cell separator, and counted using a hemocytometer. 500,000 B cells were isolated for sequencing the total repertoire. In the vaccine group, remaining B cells were labeled with Live/dead-Aqua, CD19-FiTC, CD20-APCH7, CD27-PECy7, CD38-PE, HLA-DR-PerCpCy5 and HBsAg-APC. Viable, CD19 +, CD20 +, HBsAg + B cells and viable CD19 +, CD20 −, CD27 +, CD38 +, HLA-DR + PCs were then isolated using a MoFlo cell sorter (Beckman Coulter). For competition experiments, unconjugated HBsAg was also added to the labeling mixture. Sorted cells were frozen in RLT buffer (Qiagen) at − 80 °C prior to repertoire sequencing.

### 2.5 Repertoire Sequencing

RNA was extracted from sorted cells using the RNeasy Mini Kit (Qiagen), and reverse transcription performed using SuperScript III (Invitrogen), and random hexamer primers (42 °C for 60 min, 95 °C for 10 min). PCR was conducted using the Multiplex PCR kit (Qiagen), and 200 nM VH-family specific forward primers, with IgM and IgG-specific reverse primers in separate reactions (Wu et al., 2010) (94 °C for 15 min, 30 cycles of 94 °C for 30 s, 58 °C for 90 s and 72 °C for 30 s, and 72 °C for 10 min). Amplicons were gel-extracted and purified prior to MiSeq library preparation. Samples were multiplexed, and sequenced across four 2 × 300 bp MiSeq runs.

### 2.6 Raw Sequence Processing

Sequences from each input sample were de-multiplexed, and paired ends joined using fastq-join (ea-utils). After filtering for a minimum Phred quality of 30 over 75% of bases, sequences were submitted to IMGT/HighV-Quest (Brochet et al., 2008) for annotation. There was then further filtering for reads defined as productive by IMGT. Total repertoire samples were normalized by random subsampling to 100,000 sequences per sample. Sequences from HBsAg + and PC + samples were pooled, and sequences with duplicate complementarity-determining region (CDR) 3 amino acid (AA) sequences removed.

### 2.7 Sequence Clustering

Sequences from total repertoire samples and naïve samples were clustered into clonal lineages based on CDR3 AA sequence similarity and V and J gene segment usage, using a previously described method (Galson et al., 2015). To be included in the same cluster, sequences had to have the same length CDR3 AA sequence, with no more than 1 mismatch per 12 AA's and use the same V and J gene segments. This threshold will include both clonally related sequences, and similar sequences arising from PCR error (Galson et al., 2015). Samples from all participants and timepoints were clustered together to allow easy comparison of clusters between participants and over time. The contribution of sequences to each cluster was determined separately for each sample, so that the data could subsequently be split by sample.

### 2.8 Cluster-level Annotation

For each sample, clusters were annotated for their CDR3 AA sequence length, V and J gene segment usage, the total number of sequences in the cluster, the number of unique CDR3 AA sequences in the cluster, and the average number of V gene mutations of the sequences in the cluster. The frequency of each cluster in each sample was also calculated by dividing the total number of sequences in the cluster by the total number of sequences for the sample and multiplying by 100. Clusters were defined as shared between samples if each sample contributed at least one sequence to the cluster. Sequences from HBsAg + and PC + sorted cells were then compared to clustered data to see which clusters they would fall into — these clusters were then annotated appropriately. For this comparison, the HBsAg + and PC + sequences were only matched back to participants from whom those sequences were not obtained in order to reduce the effect of non-specific matching. In addition, sequences from previously described TT-specific (Dekosky et al., 2013, Frölich et al., 2010, Lavinder et al., 2014, Poulsen et al., 2011) and influenza-specific mAb sequences were compared to the clustered data in the same way.

### 2.9 Repertoire-level Annotation

Mean CDR3 AA length and number of V gene mutations were calculated for the entire repertoire of each sample; for these calculations, clusters were weighted according to the number of sequences they contained. Repertoire diversity was calculated using Shannon's diversity index from ecology, where each cluster is considered a distinct species (Stewart et al., 1997).

### 2.10 Statistical Analysis and Graphing

Statistical analysis was performed using R (R Development Core Team, 2008), with ggplot2 (Wickham, 2009) for graphing. Principal component analysis was performed using the prcomp function in the ‘stats’ package. T-tests or Mann–Whitney U tests were used where appropriate to compare groups. Correlations were calculated with Spearman or Pearson correlation coefficients where appropriate. Correlations were graded as low (r values between 0.2–0.39), moderate (0.4–0.59), strong (0.6–0.79), and very strong (≥ 0.8).

## 3 Results

### 3.1 Serological and Cellular Measures of Vaccine Response

**Fig. 2:**
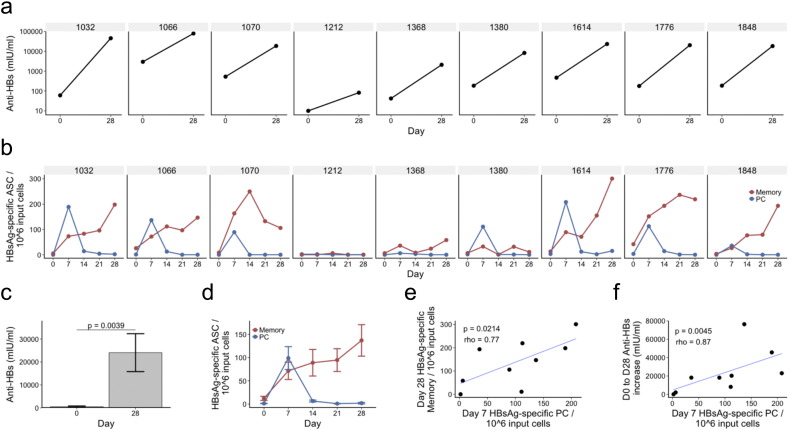
Clinical measures of vaccine response. (a) Anti-HBs antibody concentrations of each participant at day 0 and day 28 following vaccination. (b) Kinetics of HBsAg-specific memory cells and PC numbers during the study period determined by ELISpot. Input cells are PBMC's for the PC detection assay, and cultured lymphocytes for the memory cell detection assay. (c & d) Same as a and b, but displaying mean values ± SEM of the nine participants. (e) Correlation between HBsAg-specific PC numbers at day 7, and HBsAg-specific memory cell numbers at day 28. (f) Correlation between HBsAg-specific PC numbers at day 7, and the absolute increase in antibody concentration between day 0 and 28. For e and f, rho represents Spearman's rank correlation coefficient.

All nine previously vaccinated participants had an increase in anti-HBs antibody concentration to greater than 100 mIU/ml by day 28 following vaccination (p = 0.0039), There was considerable inter-individual variation in both the pre-vaccination and post-vaccination antibody concentrations (Fig. 2A/C). HBsAg-specific IgG PC numbers in the peripheral blood, as determined by ELISpot, peaked 7 days following vaccination in all participants (mean 99, range 2–208 PCs/10^6^ PBMCs), and were present at negligible numbers on all other days (Fig. 2B/D). HBsAg-specific IgG memory cell frequency, as determined by ELISpot, peaked at a later timepoint than the PCs; the greatest number were seen 28 days following vaccination (mean 72, range 0–163 memory B cells/10^6^ cultured lymphocytes). Day 7 PC numbers were strongly correlated both with day 28 memory B cell numbers (Spearman r = 0.77, p = 0.0214; Fig. 2E), and the absolute increase in antibody concentration between day 0 and day 28 (Spearman r = 0.87, p = 0.0045; Fig. 2F). The nine participants with no prior HepB vaccine history (naïve group) had no HBsAg-specific IgG memory cells or PCs detectable by ELISpot.

### 3.2 High-throughput Sequencing of the Total Immunoglobulin Repertoire

Sequence data were obtained for total IgG and IgM transcripts from all nine vaccinated participants on day 0, 7, 14 and 28, and from five participants also at day 21. On average 365,863 (range 203,151–1,023,663) raw reads were obtained per sample (Table S1). After processing and QC, this dropped to 282,432 (109,499–1,195,321) per sample, of which 100,000 per sample were randomly subsampled for normalization and then used for further analysis. This gave a total of either 1,000,000 or 800,000 total sequences for each participant depending on the availability of day 21 samples. Following clustering, there were on average 14,560 (1604–26,375) clusters per sample for the IgG dataset, and 55,612 (19,088–79,102) clusters per sample for the IgM dataset. In addition, sequence data were obtained for total IgG transcripts from the 9 participants in the naïve group, giving 312,701 (277,691–342,026) raw reads, and following subsampling to 100,000 filtered reads, 11,605 (4712–22,701) clusters for each sample (Table S2).

Samples were then characterized based on the total repertoire metrics of diversity (calculated using Shannon's diversity index), average V gene mutation, and average CDR3 AA sequence length; these are metrics previously noted to be associated with the degree of differentiation of B cell populations, and were used for initial sample quality control and outlier detection (Galson et al., 2015). Principal component analysis (PCA) based on these metrics showed that IgG and IgM samples clustered independently, but IgG samples from the naïve group did not appear different to IgG samples from the vaccinated group (Fig. S1). However, most day 7 IgG samples in the vaccinated group had distinct features compared to the rest of the IgG samples; this was not so apparent in the IgM dataset. The day 0 IgG and IgM samples from participant 1368 appeared to be outliers. In addition, all IgM samples from participant 1070 were distinct from the rest of the IgM samples, and appeared to cluster more with the IgG samples.

### 3.3 Repertoire Expansions are Greatest 7 days Post-vaccination, and Correlate with PC Numbers

**Fig. 3:**
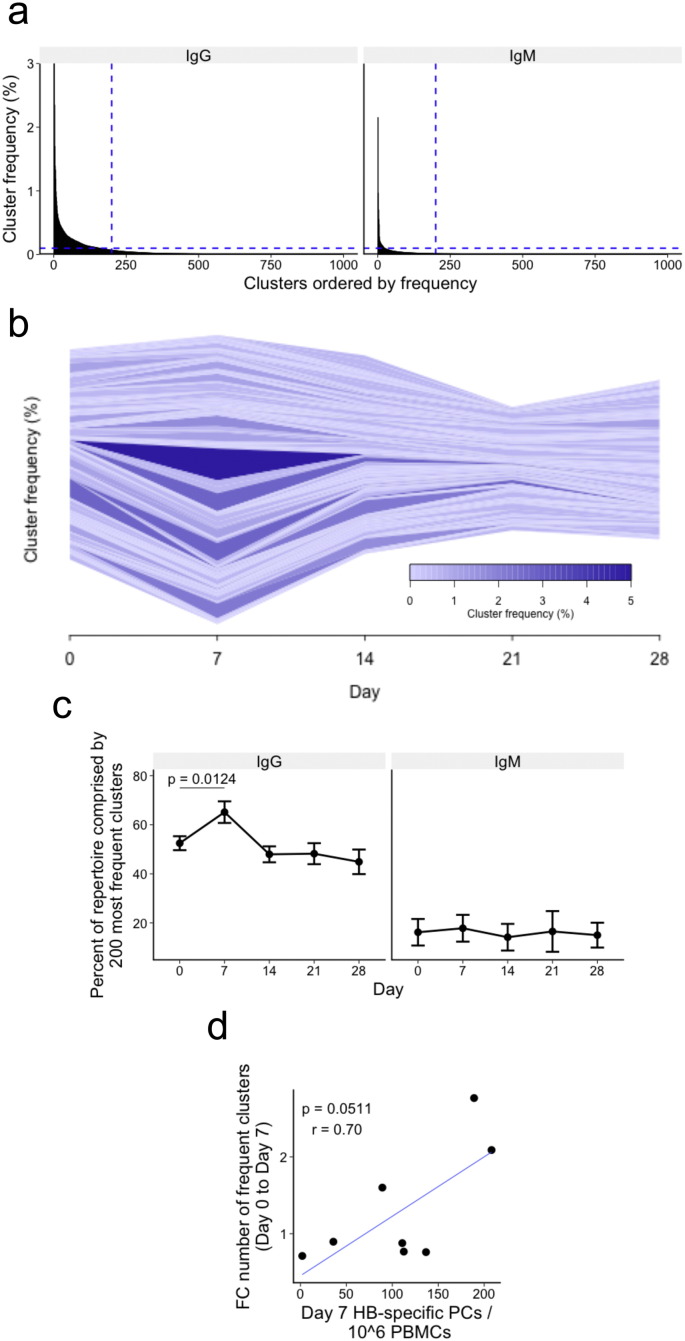
Day 7 repertoire expansions. (a) Histogram of the most frequent 1000 clusters ordered by frequency. Bars represent mean values of samples from all 41 samples. Vertical lines cross the x-axis at cluster number 200, and horizontal lines cross the y-axis at 0.1% (representing clusters containing 100 sequences). (b) Representative cluster kinetics plot from participant 1066 for their IgG repertoire (see Fig. S2 for plots from all participants). The 200 most frequent clusters at each day were found for this participant, and the frequencies of these clusters were plotted as a stacked bar chart, centered to the middle of the y-axis at each day. Clusters from each day were then joined using a horizontal stream to illustrate how the frequency of the clusters changes over time. The width and darkness of the stream represents the frequency of the cluster at that time. (c) Mean percent of the total repertoire comprised by the top 200 most frequent clusters at each day. Error bars indicate ± SEM from 8 participants. P value represents the result from a t-test. (d) Correlation between day 7 PC numbers, and the fold change in the number of clusters present at a frequency greater than 0.1%. r represents the Pearson product–moment correlation coefficient.

To visualize the degree of clonal expansion in the repertoire, the top 200 most frequent clusters were first selected from each sample. Taking the top 200 clusters is a conservative method to include all expanded clusters in the repertoire present at a frequency greater than 0.1% (Fig. 3A). Determining the frequencies of these clusters at each timepoint allowed us to track their kinetics over the course of the study (Figs. 3B & S2). Although there was great inter-individual variability in the kinetics of their large clusters, nearly all participants had a degree of expansion 7 days following vaccination. In general, day 7 cluster expansions were more pronounced in IgG than IgM repertoires. Of note, the repertoire of participant 1368 appeared to contain a large number of clonal expansions on the day of vaccination, and the repertoire from participant 1070 had a number of large expanded clusters that were present at all timepoints both in the IgG and IgM dataset (Fig. S2). Both datasets also contained a single expanded cluster present at a frequency greater than 10% at all timepoints (although the identification of this cluster was different in the IgG compared to IgM dataset). Based on the finding of clonal expansions at day 0, and being an outlier by PCA, participant 1368 was excluded from downstream analysis due to suspected immune activation at day 0.

To quantify the degree of expansion in each sample, we then found the top 200 most frequent clusters from each timepoint individually for each participant. The proportion of the total repertoire the 200 largest clusters accounted for was then calculated (Fig. 3C). Although the top 200 clusters comprise only a small percent of the total number of clusters (mean 1.3% for IgG and 0.4% for IgM), sequences contained in these clusters account for a large percentage of the total repertoire (mean 55% for IgG and 16% for IgM). The percentage of the repertoire comprised by these expanded clusters increased 7 days following vaccination (p = 0.0124) for the IgG dataset, and then decreased back to a baseline-like state by 14 days following vaccination. There was no significant change in the percent of the repertoire comprised by these expanded clusters at any day in the IgM dataset. The fold change in the number of expanded clusters (present at a frequency greater than 0.1%) between day 0 and day 7 was strongly correlated, with the number of HBsAg-specific PCs in the peripheral blood 7 days following vaccination (r = 0.70, p = 0.0511; Fig. 3D).

### 3.4 Day 7 Repertoire Expansions Impact Total Repertoire Properties

**Fig. 4:**
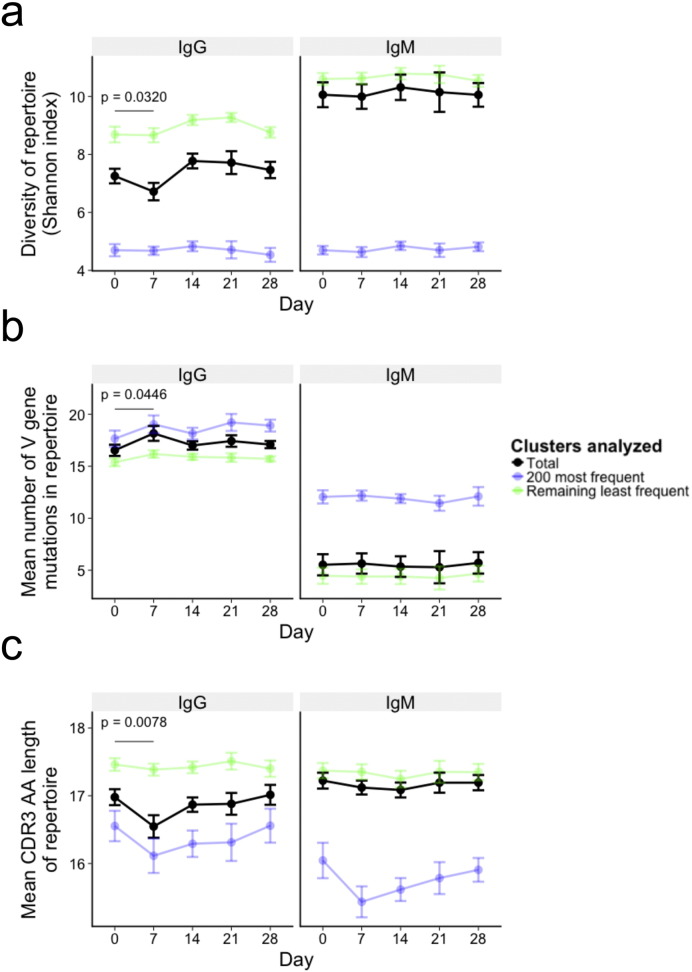
Expansion-induced changes in diversity, mutation, and CDR3 AA length. Changes in mean repertoire diversity (measured using Shannon's index with each cluster considered a distinct species) (a), mean number of V gene mutations from all sequences in the repertoire (b), and mean CDR3 AA sequence length from all sequences in the repertoire (c). Mean ± SEM shown for 8 participants; black points are values from the total repertoire, green are from sequences contained within the 200 most frequent clusters from that sample, and blue are from the remaining clusters. P value represents the result from a t-test comparing day 0 and day 7 values from the total repertoire.

Based on the total repertoire metrics of diversity, average V gene mutation, and average CDR3 AA sequence length, IgM samples were more diverse, less mutated, and had longer CDR3 AA sequences compared to IgG samples (Fig. 4A–C, black). There were no significant changes in these features over the course of the study for the IgM dataset. For the IgG dataset, the repertoire became less diverse (p = 0.0320), more mutated (p = 0.0446), and had a shorter average CDR3 AA length (p = 0.0078) at day 7 compared to day 0 (Figs. 4A–C & S1), consistent with increased B cell stimulation at day 7. There were no differences between day 0 and any of the other timepoints.

The dataset for each sample was then split into the 200 most frequent clusters, and the remaining clusters, and the total repertoire metrics calculated for the split dataset (Fig. 4A–C, blue and green). The 200 most frequent clusters were less diverse, more mutated, and had a shorter average CDR3 AA length than the remaining clusters. In addition, the 200 most frequent clusters for the IgG samples became even less diverse, more mutated, and had shorter CDR3 AA length at day 7 compared to day 0; this difference between day 0 and day 7 was not seen for the remaining less frequent clusters. It therefore appears to be the frequent clusters that drive the changes seen in the total repertoire at day 7, but even without enriching for these frequent clusters it is still possible to determine changes in the metrics of the total repertoire at day 7.

### 3.5 Repertoire Convergence Between Individuals Peaks 14–21 days After Vaccination

**Fig. 5:**
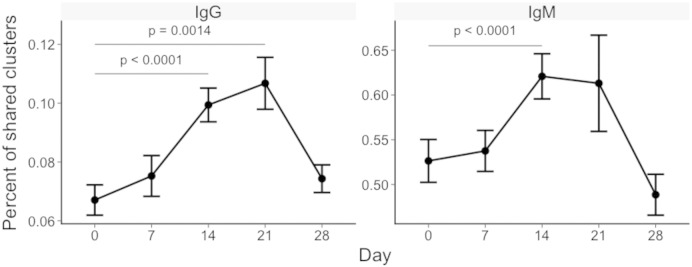
Repertoire convergence. At each day, the percent of clusters shared by each pair of two participants was determined (8 participants, giving 28 different pairings). Shown are the mean values ± SEM of the percent of clusters shared between each pair. Percent is calculated as (A ∩ B/sum(A,B) ∗ 100. P values represents the result from a t-test comparing day 0 and day 7 values from the total repertoire.

To investigate repertoire convergence following vaccination, we compared every participant to every other participant to see how many clusters were shared between each pair of two participants (Fig. 5). This was relatively low at day 0 (mean of 20 (0.07%) for IgG and 601 (0.53%) for IgM). The number increased following vaccination, and peaked at day 21 (mean of 45 (0.11%) for IgG and 806 (0.61%) for IgM). For IgM, the increase in sharing from day 0 was only significant for day 14 (p < 0.0001), but for IgG the increase in sharing was significant for days 14 (p = 0.0014) and 21 (p < 0.0001). Shared clusters were more mutated (for IgG only), had shorter CDR3 AA regions, and comprised a larger proportion of the total repertoire than the clusters that were unique to a single participant (Fig. S3).

### 3.6 Generating a Database of Enriched Vaccine Antigen-specific Clusters

**Fig. 6:**
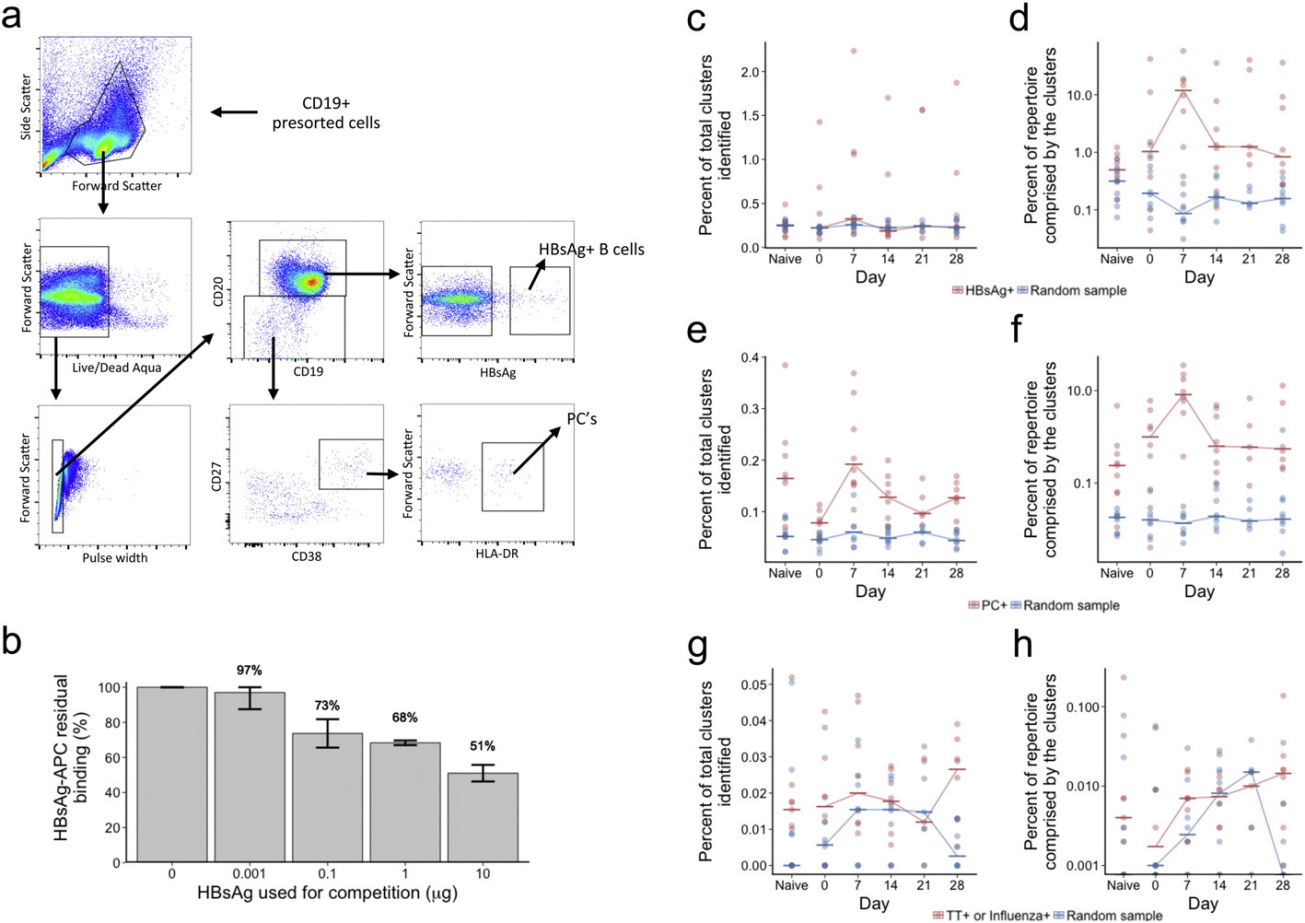
Kinetics of antigen-specific clusters. (a) FACS gating strategy for isolating HBsAg + B cells and PCs from CD19 + enriched cells. (b) Results from competition experiment where unconjugated HBsAg was added to the antibody mix at the same time as the HBsAg-APC. Mean and ± SEM shown for three tests of each condition. (c) Of the 1360 total clusters annotated as HBsAg + based on the sequence data from sorted cells, the number of these in each participant at each day was determined, and expressed as a percentage of the total number of clusters present in that participant at that day. In addition, a random sample of 1360 clusters was also taken from the dataset, and the same calculation done as a control. (d) Percent of the total repertoire comprised by the clusters identified in a (ie, corrected for cluster size). (e & f) show the same as c & d, but with the 289 clusters annotated as PC +. (g & h) show the same as c & d, but with the 65 clusters identified in our dataset that matched to previously described sequences specific for either TT or influenza. For all plots, horizontal bars show the median value from either the 8 vaccinated participants or the 9 naïve participants.

We used a fluorescence-activated cell sorting (FACS) approach, using HBsAg conjugated to APC, to isolate and sequence HBsAg + B cells (Fig. 6A). The specificity of staining was shown to be at least 50% by competition with unconjugated HBsAg (Fig. 6B). In total, 85,000 HBsAg + B cells were isolated after vaccination from five of the participants included in this study (Table S3). FACS was also used to isolate a total of 3444 PCs at day 7 (PC +) from the same five participants (Table S4), on the basis that these are likely to be significantly enriched for HBsAg specificity. Sequences from these cells were then matched back to the total IgG repertoire of both the vaccinated and naïve participants, and clusters annotated as HBsAg + or PC + based on whether sequences from the sorted cell datasets matched to them. To reduce the effect of sequences from non-specifically stained cells, sequences were only matched back to participants from whom those sequences were not obtained. The probability of any two individuals sharing a given randomly selected sequence is very low, so sequences are unlikely to match to the repertoire of a different individual unless they are specific to the common stimulus (HBsAg). This matching technique therefore provides an additional method for enriching for vaccine-specificity within the stained cells. Of the 482,849 total clusters in the vaccinated participants, 1129 were annotated as HBsAg + clusters and 235 were annotated as PC + clusters. In the naïve participants, there were 104,473 total clusters, of which 151 were annotated as HBsAg +, and 101 as PC +. In total, there were 63 clusters that were annotated as both the HBsAg + and PC +, which is significantly more than expected by chance (fishers exact test; p < 0.0001).

In addition to the vaccine antigen-specific HBsAg + and PC + clusters identified by FACS, a database of sequences specific for antigens unrelated to the HepB vaccine antigen was generated as a control dataset by a literature search. Tetanus toxoid (TT) and influenza-specific sequences were chosen, as there is the largest body of public data for these sequences. Sixteen influenza-specific sequences, and 32 TT-specific sequences were found that mapped to clusters in our dataset (Table S5).

### 3.7 Vaccine Antigen-specific Clusters Increase in Number and Size After Vaccination

Based on the database of enriched antigen-specific clusters, HBsAg +, PC + and TT + or influenza + clusters were searched for in each sample. In addition to these antigen-specific clusters, we also generated sets of randomly selected clusters (including the same number of clusters as the antigen-specific sets) from our IgG repertoire data to be used as a control. There was a peak in the percent of total clusters annotated as either HBsAg + or PC + at day 7 following vaccination (Fig. 6C & D). This was more pronounced for the PC + clusters than the HBsAg + clusters, and not seen when searching for the randomly sampled set of clusters. Correcting for cluster size showed an even more marked peak at day 7 for both the HBsAg + and PC + clusters (Fig. 6E & F). There were no distinct changes in the numbers of irrelevant TT + or influenza + antigens following vaccination (Fig. 6G & H), although the numbers of these clusters identified was low at all timepoints due to limitations in the number of previously published sequences we could find.

Despite considerable inter-individual variation, the clusters annotated as HBsAg + or PC + in the previously vaccinated participants were more mutated, had greater frequency, and had shorter CDR3 sequences than those not annotated (Fig. S4). In the naïve participants, there was no difference between the HBsAg + and non-annotated clusters in mutation or frequency, but the HBsAg + clusters still had significantly shorter CDR3 length than the non-annotated clusters.

### 3.8 Bioinformatic Enrichment of Vaccine Antigen-specific Clusters from the Total Repertoire

**Fig. 7:**
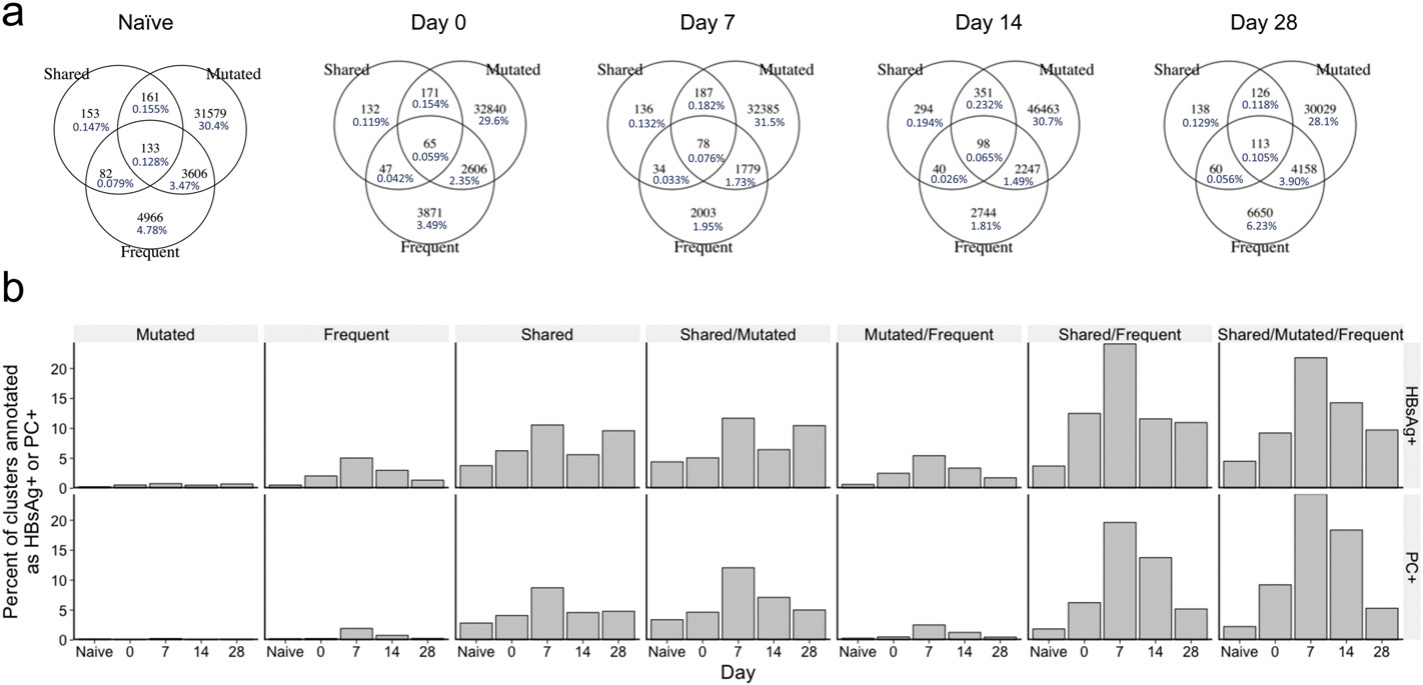
Bioinformatic enrichment of vaccine-specific clusters from the total repertoire. (a) Venn diagrams showing the overlap in the number of the shared (by more than two participants), mutated (more than 18 V gene mutations on average), and frequent (more than 0.01% of the total repertoire) clusters at each day from the 8 vaccinated participants, and also the 9 naïve participants. Day 21 is omitted, as there were no samples from four of the participants at this timepoint. Percentages are calculated by dividing the number of clusters present in a particular category by the total number of clusters at that timepoint. (b) In each category in the Venn diagrams, the percent of the sequences that were annotated as HBsAg + or PC + based on the FACS data was determined.

Based on the total repertoire signatures observed, and the properties of the vaccine antigen-specific clusters, we developed a model to enrich for vaccine-activated clusters (i.e., likely to be HBsAg-specific, but could potentially be specific to an irrelevant antigen due to bystander activation) from the total repertoire. We focused on the IgG dataset, as the signals were stronger, and class-switched cells dominate the response to booster vaccination (Blanchard-Rohner et al., 2009). From the data analysis and a priori consideration of features likely to be associated with a response to vaccination we used the properties of cluster frequency, mutation number and cluster sharing to develop a model to enrich for vaccine antigen-specific clusters. The model did not require having a short CDR3 sequence, as this may not be a universal property of all antigens, so not useful outside of the context of this specific vaccine. Additionally, the HBsAg + and PC + clusters present in the naïve participants were also short, so this may just be due to chance. Conservative cutoff values of more than 18 mutations, and a frequency greater than 0.01% (i.e. minimum of 10 sequences in the cluster) were set based on the distributions of these variables (Fig. S5). Searching for clusters that fit all three requirements on each day showed that there were 133 present in the naïve participants compared to 65 present in the previously vaccinated participants at day 0 (Fig. 7A); however, these populations are not directly comparable, as there were nine participants in the naïve group compared to eight in the previously vaccinated group, so there will be more shared sequences by chance in the naïve group. The number of clusters that fit all three requirements increased at each successive visit following vaccination up to 114 clusters at day 28.

To validate our model for identifying clusters likely to contain vaccine-specific sequences, we compared the clusters identified in the model to those annotated as HBsAg + or PC + based on the FACS data (Fig. 6B). This was done separately for each metric in the model. By combining the metrics of sharing, mutation, and frequency, it was possible to obtain greater enrichment for vaccine-specific clusters than using either of these metrics on their own. Despite identifying more clusters that fitted the model parameters in the naïve group, these were less enriched for HBsAg + or PC + specificity than those from the vaccinated group even at day 0 (9.2% HBsAg + and 9.2% PC + in the vaccinated group at day 0 compared to 4.5% and 2.3% respectively in the naïve group). The greatest enrichment was seen at day 7, where 22% of the 78 enriched clusters were annotated as HBsAg + and 24% annotated as PC +. The model therefore gave 42 × enrichment for HBsAg + clusters, and 168 × enrichment for PC + clusters at day 7, compared to looking at total clusters. Following day 7, the degree of enrichment decreased at each subsequent timepoint sampled. Considering each metric independently, shared clusters contained the highest percentage of HBsAg + and PC + clusters, followed by frequent clusters, and finally mutated clusters. The kinetic of the enrichment seen in the shared clusters was also slightly different to that of mutated or frequent clusters, as there was an increase seen again at day 28.

## 4 Discussion

Using HepB booster vaccination as a model system, we applied in-depth sequence analysis of the immunoglobulin heavy chain repertoire from circulating B cells to thoroughly characterize how the B cell repertoire responds to secondary antigen encounter. Although antigen-specific B cells account for only a small proportion of the total peripheral B cells even at the peak of their appearance, we were able to detect perturbations in global repertoire properties 7 days following vaccination. These changes were characterized by a decrease in diversity, an increase in mutation, and a decrease in CDR3 sequence length — features that appear to be generally characteristic of more activated B cells (Galson et al., 2015), and can be related to the appearance of vaccine-induced PCs in the peripheral blood at this time. These changes were only seen in the IgG dataset, which is to be expected, as this is a secondary response so will be stimulating switched memory cells. Interestingly, despite these perturbations being most pronounced 7 days after vaccination, the greatest sequence similarity between the repertoires of different individuals was seen on days 14 and 21 after vaccination.

Although day 7 repertoire perturbations have previously been observed (Jackson et al., 2014, Laserson et al., 2014, Wang et al., 2014), to our knowledge the repertoire similarity on days 14 and 21 have not been previously studied. The minimal repertoire overlap we observe at day 0 is to be expected considering the enormous diversity of the repertoire, and our sampling depth of 100,000 reads (Boyd et al., 2009, Galson, 2015). Given the diversity of the immunoglobulin repertoire, the finding of two identical CDR3 AA sequences in 2 different individuals is unlikely to occur by chance alone (Saada et al., 2007), so could be indicative of common selection pressures on the repertoire of different individuals. The increase in convergence we see following vaccination is in line with the vaccine antigen either imposing such a selective pressure, causing convergent evolution of the repertoire in the different individuals to produce sequences specific for the antigen, or simply expanding pre-existing memory cells that have already been selected in this way. Such antigen-driven convergence has been seen following immunization with highly repetitive polysaccharide antigens (Trück et al., 2015), and also in the context of influenza vaccination (Jackson et al., 2014). Both of these studies investigated convergence 7 days following vaccination, as this coincides with the peak of PCs in the peripheral blood in a secondary response, so is expected to give the greatest signal. Our study confirms that convergence is also seen in the context of a simple protein antigen (with Alum adjuvant), but by sampling at more timepoints, shows that the increase in convergence at day 7 is relatively minor, and instead the most significant signals are seen on days 14 and 21. The kinetics of the increase in convergence is therefore more in line with the kinetics of vaccine-specific memory cells in the peripheral blood rather than PCs. One interpretation of this is that following vaccination there is rapid activation of circulating memory cells to terminally differentiate into low-affinity PCs by day 7, as demonstrated by the greatest repertoire expansions seen in our study. However, it is likely that there is also formation of germinal centers, which mediate further proliferation and selection for antigen binding, and the production of higher-affinity memory cells at later timepoints (Bachmann et al., 1996, Mcheyzer-williams et al., 2015). The small increase in convergence at day 7 may therefore be a result of low-affinity PC sequences, while the increased convergence later on may be a result of the higher-affinity memory cell sequences. Although we do not see an increase in convergence at day 28 when there are the greatest numbers of circulating memory cells detectable by ELISpot, this may be because the circulating memory cells are all clonally related by this time, so sequences derived from these cells would all be incorporated into a single sequence cluster. Alternatively, this may be an artifact of the stimulation method used for memory cell detection by ELISpot in that the day 28 memory cells are more readily stimulated to secrete antibody than earlier memory cells. Nevertheless, these results suggest that careful consideration of timepoints should be taken when using immunoglobulin repertoire sequencing to find mAb sequences. Normally, day 7 PCs are isolated and sequenced, but the resulting mAbs can be of low affinity (Reddy et al., 2010, Wrammert et al., 2008); if later timepoints were instead chosen and the memory cell sequences identified, it may be possible to generate higher affinity mAbs.

By sorting and sequencing HBsAg + (memory) and PC + cells, we were able to confirm that cells specific to the vaccine caused the signatures we saw in the repertoire. Both the HBsAg + and PC + clusters had increased convergence, mutation, and size compared to the total repertoire, and tracking the kinetics of these clusters showed the greatest peak at day 7. Based on our observations of what comprises a vaccine-specific cluster, we were able to create a simple model for de novo identification of such clusters from the total repertoire. Although cluster expansion alone appears to be sufficient to identify likely day 7 PC clusters, adding in the metrics of cluster sharing and cluster mutation improved enrichment of vaccine-specific clusters present at other timepoints. It may also be possible to identify different types of cells (memory cells vs PCs) by considering different metrics. By looking at whether the clusters identified were indeed annotated as HBsAg + or PC + by comparison to sequences from the sorted cells, we were able to validate the model.

We are unable to determine specificity of the model, as we will not have sorted and sequenced the entire vaccine antigen-specific repertoire, so there may be more clusters in the enriched population that are vaccine antigen-specific, but were not annotated as such. An additional limitation was that the HBsAg + cell sorting only had approximately 50% specificity. Furthermore, for the PC + cell sorting, although we focused on HLA-DR + PCs, as these are a recently activated population that will be enriched for vaccine-specificity (Odendahl et al., 2005), we cannot rule out that some of the PCs will have alternate specificities. Indeed, this appears quite likely, as even the naïve participants had clusters annotated as HBsAg + and PC +. However, by only matching the sequences back to the repertoire of individuals from whom the sequences were not obtained, this specificity should be increased, as the sequences are unlikely to match to the repertoire of a different individual unless they are specific to the common antigen. We are unable to clone and functionally confirm the specificity of these sequences, as we are just sequencing the heavy chain, so have no paired light chain sequences to express them with. Currently paired heavy:light chain sequencing is technically challenging to perform in high throughput, although since conducting this study, there have been significant advances in this area, offering promise for future work (DeKosky et al., 2015).

Despite the limitations, we show that with sufficiently stringent parameters, it appears possible to enrich for antigen-specific sequences in populations that have encountered a common antigen, and that this is possible at least 28 days following antigen encounter. However, it must also be noted that the model also enriched for HBsAg-specific clusters at baseline before the participants had received the common stimulus. This may be due to all participants having been previously immunized with HepB vaccine, making it possible to detect historic HBsAg-specific memory cells. Indeed, this signal appears greater in individuals previously immunized with HepB, compared to the unimmunized naïve group.

The ability to de novo enrich for antigen-specific sequences (even if not 100% specifically), has important clinical implications (Galson et al., 2014). In the context of vaccination, identifying the responding sequences can be used to identify mAbs as previously mentioned. In addition, numbers of these sequences could potentially be used for novel correlates of vaccine-mediated immunity (Trück et al., 2015); although we could correlate expanded clusters with PC numbers in this study, studies with larger numbers of participants are required to further validate this. In the context of disease, in many cases, there are no definitive diagnostic tests. If common sequences or repertoire signatures could be identified between individuals with certain diseases, these could be used as diagnostic candidates. Here, it is necessary that the signatures in the repertoire be persistent, and not limited to day 7 following antigen encounters.

Whilst we were able to identify common signatures that were associated with a robust B cell response and could be used to identify vaccine-specific clusters, there was considerable inter-individual variation in the global repertoire properties, and response dynamics in different individuals, which can give tailored insight into individual responses. For example, there was one participant (1212) who did not have a peak of PCs appearing in the peripheral blood at day 7, but did have an increase in anti-HBs in the blood, so must have produced vaccine-specific PCs at some time during the study period. Sampling at day 7 may have missed the PC burst in the peripheral blood (Henn et al., 2013), or we may have been limited by the sensitivity of the ELISpot assay. At the sequence level, there were clear day 7 clonal expansions in this participant, and these expansions were actually greater than in other participants. It could be the case therefore that this participant just produced a small number of PCs (undetectable by ELISpot), but that these PCs produced large amounts of antibody (consistent with the large clonal expansions). In addition, we noticed in one participant (1070), that there was a highly expanded cluster (> 10% of total repertoire) present at every timepoint in both the IgG and IgM dataset. We can speculate that such an expansion may be caused by chronic infection (Wang et al., 2013) such as from cytomegalovirus, and although this was not formally tested, it highlights the additional immunological insights that can be gained from this sequencing technology. We note that analysis of the enriched vaccine-specific sequence repertoire of this participant revealed one of the strongest day 7 signals, which is interesting in light of recent work suggesting some chronic infections may enhance the immune response to vaccination (Furman et al., 2015).

To summarize, we have used immunoglobulin heavy chain repertoire sequencing, for a thorough dissection of the post-vaccination B cell response. We show day 7 repertoire signatures which appear to correlate with the PCs appearing at this time, and we show repertoire convergence increasing up to day 21 following vaccination, which has a more similar kinetic to appearance of memory cells. Measuring such signatures can give a detailed insight of an individual's response to vaccination, and may be applicable to studies of vaccine immunogenicity and function. In addition, the knowledge of these signatures allowed the development of a simple model to enrich for vaccine-specific clusters from the total repertoire. Such ability to identify sequences of importance in the response is key for fully utilizing the potential of this technology to understand immune responses to disease and vaccination.

## Conflicts of interest

R.V.D.M is an employee and shareholder of the GSK group of companies.

## Funding

Study funding was provided partly by the BBSRC and GlaxoSmithKline Biologicals SA in the form of an iCASE studentship awarded to J. D. G. (BB/J500355/1), and partly by the NIHR Oxford Biomedical Research Centre. Purified HBsAg was provided by GlaxoSmithKline Biologicals SA, and conjugated to APC by Miltenyi Biotec. Sequence data was generated by the High Throughput Genomics Group at the Wellcome Trust Centre for Human Genetics, which is subsidized by Wellcome trust grant reference 090532/Z/09/Z. A.J.P. is a Jenner Investigator and James Martin Senior Fellow. D.F.K. receives salary support from the NIHR Oxford Biomedical Research Centre.

## Author contributions

A.J.P, D.F.K, E.A.C, J.T, J.D.G, V.C. & R.V.D.M conceived the study. J.D.G & E.A.C collected data. J.D.G performed data analysis. A.F., M.M., & G.L assisted with bioinformatic work. C.R. conjugated APC to HBsAg. J.D.G., J.T., E.A.C, A.J.P & D.F.K interpreted data. J.D.G. wrote the manuscript, with input from all authors.
