# Supplementary material for: Analysis of B Cell Repertoire Dynamics Following Hepatitis B Vaccination in Humans, and Enrichment of Vaccine-specific Antibody Sequences
Source: eBioMedicine. 2015 Nov 24;2(12):2070–9. doi: 10.1016/j.ebiom.2015.11.034 (PMC4703725; doi:10.1016/j.ebiom.2015.11.034)
Supplement: Supplementary file 1 — Supplementary material [file mmc1.pdf]

Supplementary Information

Analysis of B cell Repertoire Dynamics Following Hepatitis B Vaccination in Humans, and Enrichment of Vaccine-specific Antibody Sequences

Jacob D. Galson[a,b], Johannes Trück[a,c], Anna Fowler[b], Elizabeth A. Clutterbuck[a], Márton Münz[b], Vincenzo Cerundolo[d], Claudia Reinhard[e], Robbert van der Most[f], Andrew J. Pollard[a], Gerton Lunter[b] & Dominic F. Kelly[a]

[a] Oxford Vaccine Group, Department of Paediatrics, University of Oxford and the NIHR Oxford Biomedical Research Center, Oxford, OX3 7LE, United Kingdom. [b] Welcome Trust Centre for Human Genetics, University of Oxford, Oxford, OX3 7BN, United Kingdom. [c] Paediatric Immunology, University Children's Hospital, Zürich, 8032, Switzerland. [d] Medical Research Council Human Immunology Unit, Weatherall Institute of Molecular Medicine, Oxford, OX3 9DS, United Kingdom. [e] Miltenyi Biotec, Bergisch Gladbach, Germany. [f] GSK Vaccines, Rixensart, Belgium

Contents

Supplementary figures:

- Fig. S1. PCA of repertoire diversity, mutation and CDR3 AA length.
- Fig. S2. Cluster kinetics plots.
- Fig. S3. Properties of shared clusters.
- Fig. S4. Properties of HBsAg+ and PC+ clusters.
- Fig. S5. Mutation and frequency distribution of clusters.

Supplementary tables:

- Table S1. Summary of sequence data obtained from the previously vaccinated participants who were given the HepB booster vaccine
- Table S2. Summary of sequence data obtained from HepB naive participants.
- Table S3. Summary of HBsAg-enriched sequence data obtained following HepB booster vaccination of previously vaccinated participants.
- Table S4. Summary of PC-enriched sequence data obtained in this study following HepB booster vaccination of previously vaccinated participants.
- Table S5. Antigen-specific sequences collated from the literature that map to clusters in our dataset.

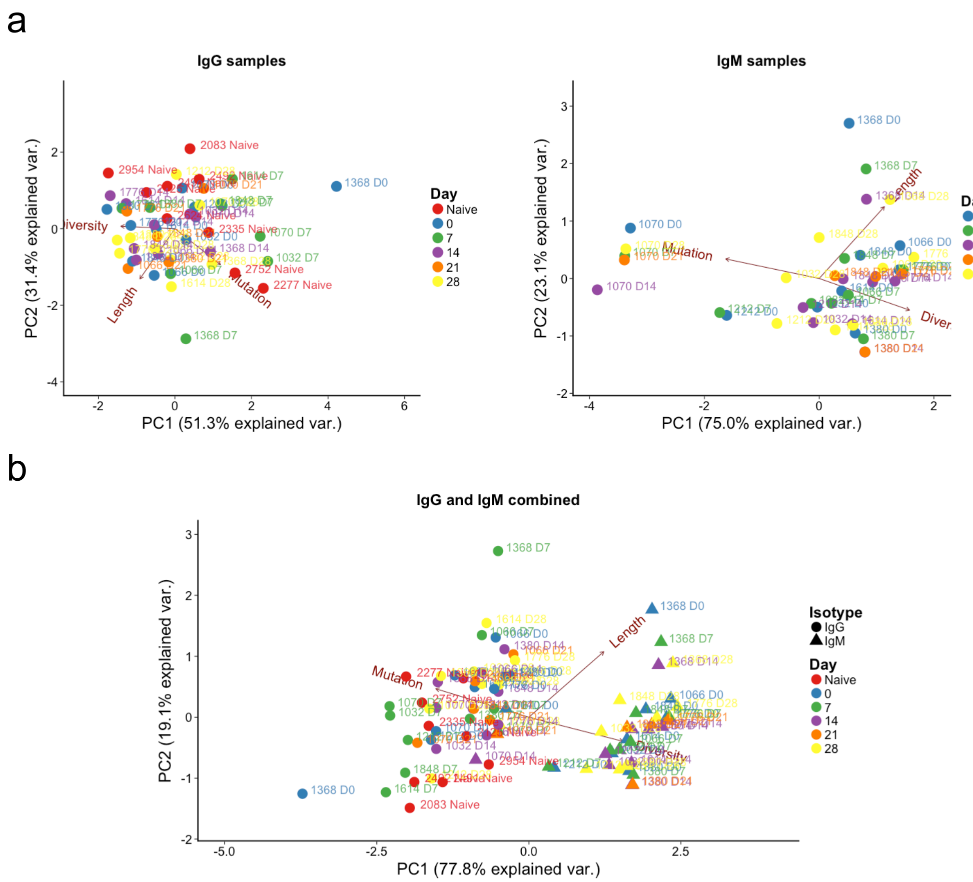

Fig. S1. PCA of repertoire diversity, mutation and CDR3 AA length. For each sample, repertoire diversity, average V gene mutation, and average CDR3 AA sequence length was determined. These were then used in a principal component analysis to detect outliers. IgG and IgM samples were either considered separately (a), or together (b).

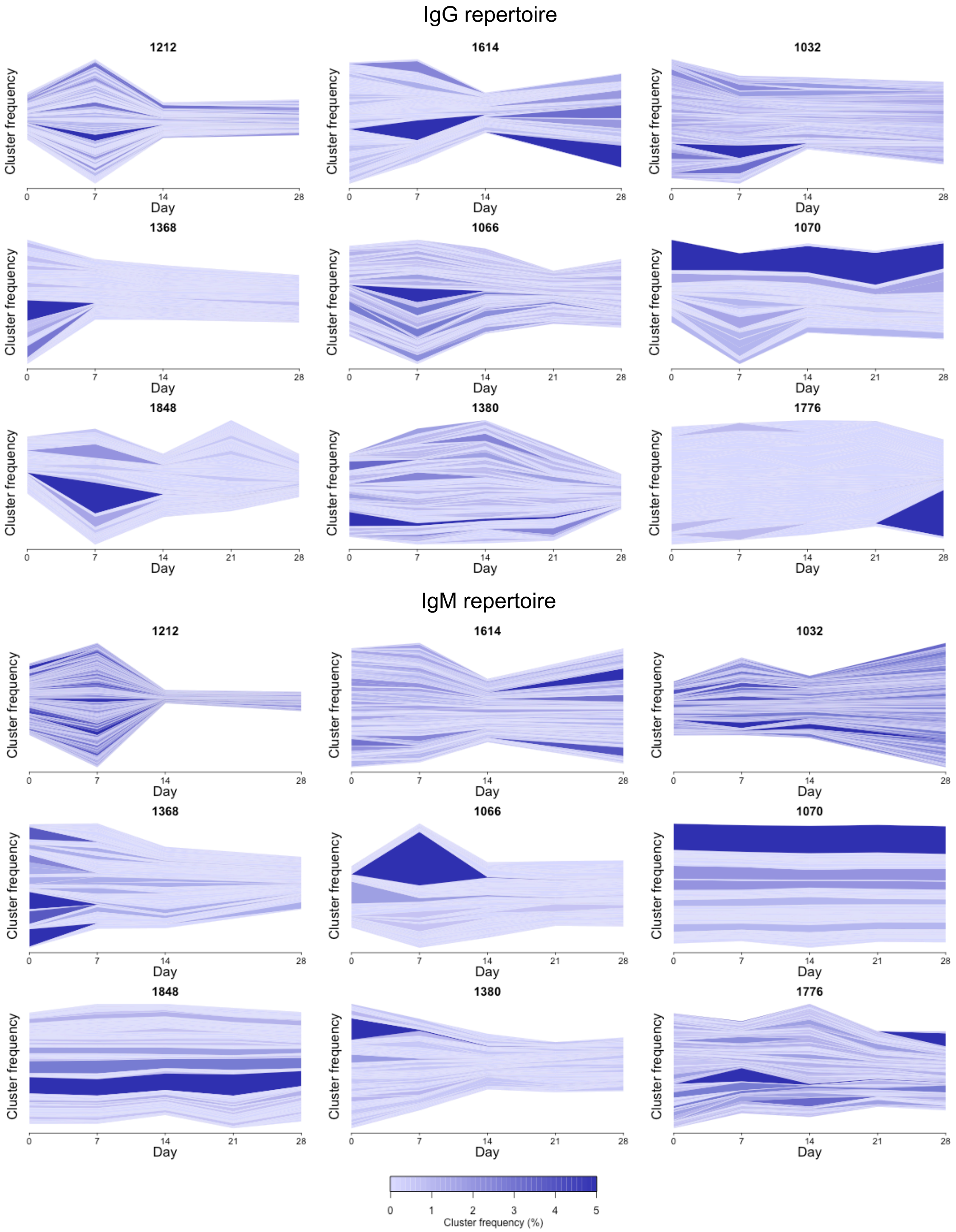

Fig. S2. Cluster kinetics plots. For each participant, the 200 most frequent clusters are found for each sample. At each day, the frequencies of these clusters are plotted as a stacked bar chart, centered to the middle of the y axis. Clusters from each day are then joined using a horizontal stream to illustrate how the frequency of the clusters changes over time. The width and darkness of the stream represents the frequency of the cluster at that time. The top 9 plots are for IgG clusters, and the bottom 9 plots are for IgM clusters.

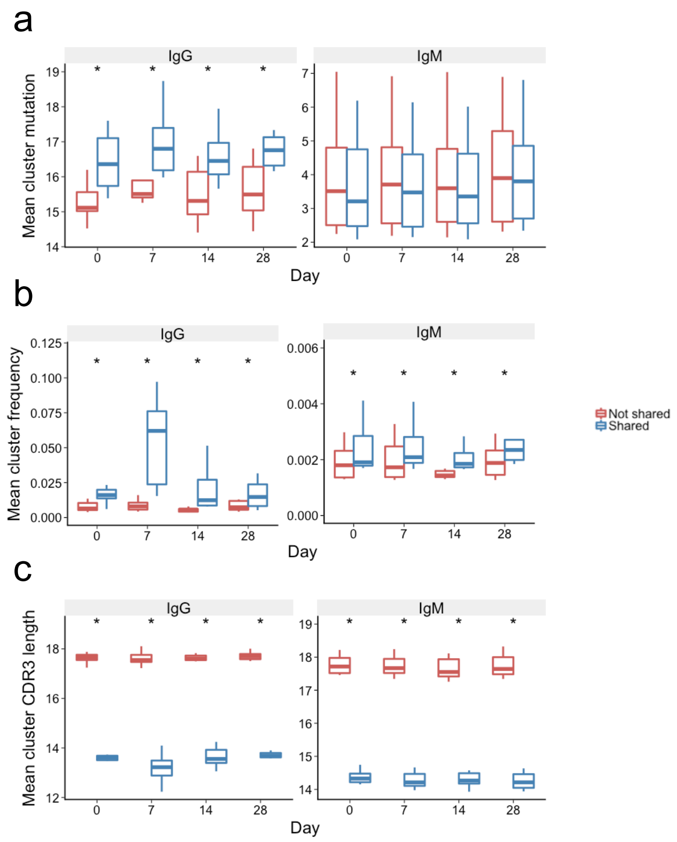

Fig. S3. Properties of shared clusters. (a) Mean number of mutations of each cluster for each participant (n=8), split by clusters present in more than one participant (shared) versus those present in only one participant (not shared). (b) Mean frequency of each cluster for each participant. (c) Mean CDR3 AA sequence length of each cluster for each participant. Boxes show locations of 25, 50, and 75th percentiles, and whiskers show data within 1.5x the interquartile range. * $p < 0.05$ (Mann-Whitney U test).

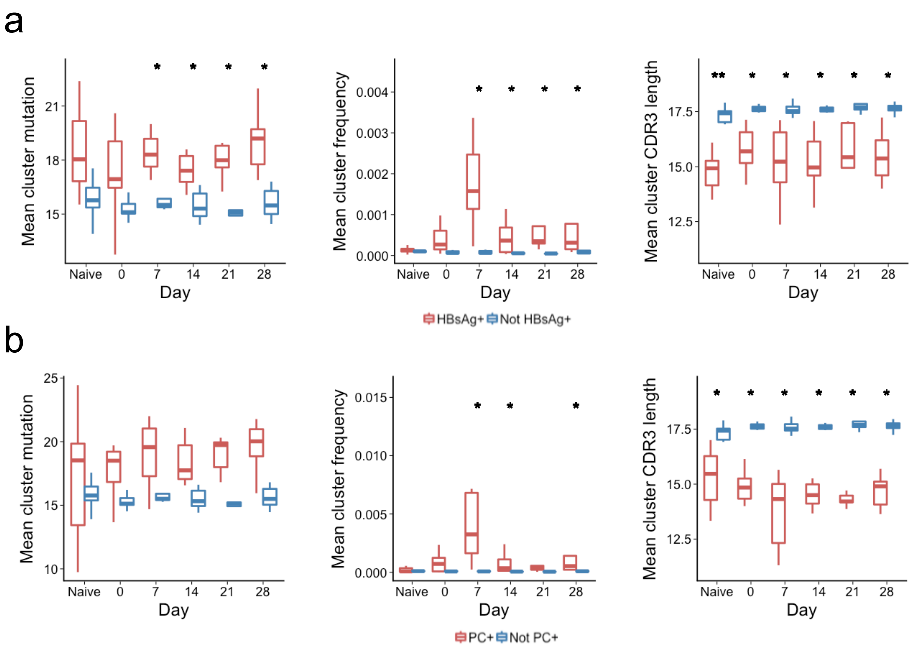

Fig. S4. Properties of HBsAg+ and PC+ clusters. For each sample, clusters were annotated as HBsAg+ (a) or PC+ (b) based on whether sequences from the HBsAg+ or PC+ FACS enriched sequence datasets matched to them. The metrics of mean cluster mutation, mean cluster frequency, and mean cluster CDR3 AA sequence length were then calculated for each sample using just the clusters annotated as HBsAg+ or PC+, and the unannotated clusters. Boxes show locations of 25, 50, and 75th percentiles, and whiskers show data within 1.5x the interquartile range. * $p < 0.05$, ** $P < 0.005$ (Mann-Whitney U test).

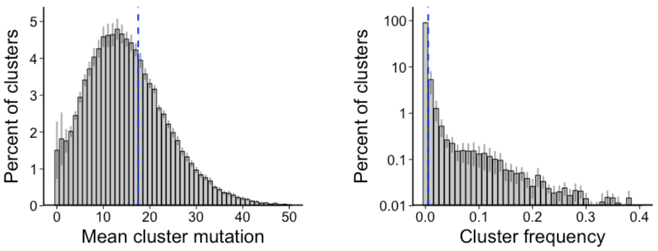

Fig. S5. Mutation and frequency distribution of clusters. Distribution of clusters in the total repertoire at day 0 according to mean number of mutations, frequency, and how many participants they are shared between. Mean values and ± SEM shown for the 8 vaccinated participants. Vertical dashed blue lines indicate where the cutoffs were chosen to be included in the model for vaccine-specific cluster enrichment.

Table S1. Summary of sequence data obtained from the previously vaccinated participants who were given the HepB booster vaccine. Only data obtained from total B cells are shown. Data from sorted HBsAg+ cells and PCs are shown in Table S3 and S4 respectively.

Participant ID	Day	B cell subset	Cell number	Isotype	Raw sequences	Filtered sequences	Clusters
1032	0	Total	500000	IgG	243879	100000	16129
1032	0	Total	500000	IgM	331231	100000	12359
1032	7	Total	500000	IgG	288204	100000	25498
1032	7	Total	500000	IgM	309031	100000	17355
1032	14	Total	500000	IgG	254121	100000	12755
1032	14	Total	500000	IgM	302460	100000	10254
1032	28	Total	500000	IgG	265032	100000	24980
1032	28	Total	500000	IgM	315870	100000	19727
1066	0	Total	500000	IgG	336302	100000	16157
1066	0	Total	500000	IgM	410635	100000	26375
1066	7	Total	500000	IgG	455339	100000	19728
1066	7	Total	500000	IgM	449097	100000	7698
1066	14	Total	500000	IgG	467586	100000	19060
1066	14	Total	500000	IgM	521114	100000	22515
1066	21	Total	500000	IgG	482029	100000	22891
1066	21	Total	500000	IgM	471378	100000	25026
1066	28	Total	500000	IgG	391338	100000	23840
1066	28	Total	500000	IgM	468921	100000	35981
1070	0	Total	500000	IgG	353992	100000	30161
1070	0	Total	500000	IgM	533009	100000	45214
1070	7	Total	500000	IgG	370941	100000	44247
1070	7	Total	500000	IgM	463595	100000	47192
1070	14	Total	500000	IgG	408608	100000	53534
1070	14	Total	500000	IgM	460813	100000	19088
1070	21	Total	500000	IgG	413979	100000	41457
1070	21	Total	500000	IgM	471874	100000	58609
1070	28	Total	500000	IgG	359743	100000	53669
1070	28	Total	500000	IgM	489340	100000	70572
1212	0	Total	500000	IgG	280407	100000	7302
1212	0	Total	500000	IgM	263810	100000	6045
1212	7	Total	500000	IgG	236620	100000	10622
1212	7	Total	500000	IgM	305753	100000	8851
1212	14	Total	500000	IgG	265090	100000	16555
1212	14	Total	500000	IgM	342949	100000	12793
1212	28	Total	500000	IgG	239019	100000	17585
1212	28	Total	500000	IgM	282469	100000	24339
1368	0	Total	500000	IgG	229626	100000	15041
1368	0	Total	500000	IgM	327464	100000	8613
1368	7	Total	500000	IgG	263905	100000	6678

1368	7	Total	500000	IgM	325264	100000	8224
1368	14	Total	500000	IgG	231683	100000	16759
1368	14	Total	500000	IgM	249160	100000	20333
1368	28	Total	500000	IgG	203151	100000	7723
1368	28	Total	500000	IgM	284665	100000	14123
1380	0	Total	500000	IgG	376162	100000	24591
1380	0	Total	500000	IgM	385190	100000	25396
1380	7	Total	500000	IgG	378682	100000	22688
1380	7	Total	500000	IgM	325063	100000	25600
1380	14	Total	500000	IgG	449052	100000	24457
1380	14	Total	500000	IgM	382603	100000	64859
1380	21	Total	500000	IgG	489708	100000	60471
1380	21	Total	500000	IgM	310318	100000	75301
1380	28	Total	500000	IgG	438162	100000	58973
1380	28	Total	500000	IgM	265220	100000	24627
1614	0	Total	500000	IgG	216878	100000	5254
1614	0	Total	500000	IgM	295468	100000	8679
1614	7	Total	500000	IgG	260694	100000	1604
1614	7	Total	500000	IgM	331460	100000	4337
1614	14	Total	500000	IgG	272195	100000	19356
1614	14	Total	500000	IgM	345007	100000	13473
1614	28	Total	500000	IgG	266968	100000	8464
1614	28	Total	500000	IgM	378363	100000	11870
1776	0	Total	500000	IgG	309604	100000	71583
1776	0	Total	500000	IgM	359614	100000	74535
1776	7	Total	500000	IgG	391962	100000	64802
1776	7	Total	500000	IgM	359678	100000	79102
1776	14	Total	500000	IgG	402395	100000	46375
1776	14	Total	500000	IgM	372711	100000	75450
1776	21	Total	500000	IgG	497248	100000	77343
1776	21	Total	500000	IgM	313332	100000	75134
1776	28	Total	500000	IgG	302880	100000	77758
1776	28	Total	500000	IgM	269306	100000	77404
1848	0	Total	500000	IgG	427766	100000	66281
1848	0	Total	500000	IgM	473752	100000	62369
1848	7	Total	500000	IgG	412862	100000	51059
1848	7	Total	500000	IgM	1023663	100000	53032
1848	14	Total	500000	IgG	458326	100000	60720
1848	14	Total	500000	IgM	329791	100000	75014
1848	21	Total	500000	IgG	439611	100000	70032
1848	21	Total	500000	IgM	508343	100000	70223
1848	28	Total	500000	IgG	295501	100000	73971
1848	28	Total	500000	IgM	464751	100000	71217

Table S2. Summary of sequence data obtained from HepB naive participants.

Participant ID	Day	B cell subset	Cell number	Isotype	Raw sequences	Filtered sequences	Clusters
2083	0	Total	500000	IgG	285665	100000	8993
2125	0	Total	500000	IgG	280250	100000	11559
2277	0	Total	500000	IgG	342026	100000	9089
2335	0	Total	500000	IgG	326107	100000	6505
2491	0	Total	500000	IgG	326473	100000	19462
2492	0	Total	200000	IgG	325226	100000	11523
2624	0	Total	120000	IgG	277691	100000	4712
2752	0	Total	500000	IgG	329479	100000	9904
2954	0	Total	500000	IgG	321396	100000	22701

Table S3. Summary of HBsAg-enriched sequence data obtained following HepB booster vaccination of previously vaccinated participants.

Participant ID	Day	B cell subset	Cell number	Isotype	Raw sequences	Filtered sequences	Unique CDR3
1066	7	HBsAg+	1760	IgG	327019	132238	2525
1066	14	HBsAg+	1359	IgG	326266	126881	3758
1066	21	HBsAg+	979	IgG	82093	35623	1859
1066	28	HBsAg+	1204	IgG	63774	29794	1637
1070	7	HBsAg+	6187	IgG	342243	121447	3539
1070	14	HBsAg+	2068	IgG	271512	102904	4421
1070	21	HBsAg+	3344	IgG	315878	115877	5237
1070	28	HBsAg+	1227	IgG	106439	47525	2291
1380	7	HBsAg+	6583	IgG	323395	108512	4175
1380	14	HBsAg+	954	IgG	62840	122900	5579
1380	21	HBsAg+	4464	IgG	297629	97685	5159
1380	28	HBsAg+	5208	IgG	35536	12839	904
1776	7	HBsAg+	8420	IgG	269573	95928	4785
1776	14	HBsAg+	15000	IgG	231163	75885	5455
1776	21	HBsAg+	6008	IgG	242903	78142	4576
1776	28	HBsAg+	9266	IgG	304594	106777	6679
1848	7	HBsAg+	1598	IgG	77476	32673	1799
1848	14	HBsAg+	6169	IgG	348983	24789	1609
1848	21	HBsAg+	1328	IgG	62811	25753	1811
1848	28	HBsAg+	1874	IgG	21648	6228	514

Table S4. Summary of PC-enriched sequence data obtained in this study following HepB booster vaccination of previously vaccinated participants.

Participant ID	Day	B cell subset	Cell number	Isotype	Raw sequences	Filtered sequences	Unique CDR3
1066	7	PC DR+	679	IgG	118301	61166	3458
1070	7	PC DR+	479	IgG	96005	49371	2119
1380	7	PC DR+	1548	IgG	118290	61766	4097
1776	7	PC DR+	333	IgG	108157	186728	2913
1848	7	PC DR+	405	IgG	113655	64510	2116

Table S5. Antigen-specific sequences collated from the literature that map to clusters in our dataset.

Antigen	Subtype	CDRH3 AA sequence	Reference
TT	NA	CATGVTLDYW	DeKosky.2013
TT	NA	CATGITLDYW	DeKosky.2013
TT	NA	CATGFTLDYW	DeKosky.2013
TT	NA	CARGVAPAGIPFDFW	DeKosky.2013
TT	NA	CARFLSGTYDYW	DeKosky.2013
TT	NA	CARDYFHSGSQYFFDYW	DeKosky.2013
TT	NA	CARQTDNWFDPW	Frolich.2010
TT	NA	CARQADNWFDPW	Frolich.2010
TT	NA	CARHADNWFDPW	Frolich.2010
TT	NA	CALTYDNWFDPW	Frolich.2010
TT	NA	CARDYFHSGSQYFFDYW	Lavinder.2014
TT	NA	CVSGSSLDYW	Poulsen.2007
TT	NA	CASGSTLDYW	Poulsen.2007
TT	NA	CARDYYGSGSHYYFDYW	Poulsen.2007
TT	NA	CARRYDFWSGFLDYW	Poulsen.2007
TT	NA	CARRHYCSSTSCYDAFDIW	Poulsen.2007
TT	NA	CARIVGTHGFDYW	Poulsen.2007
TT	NA	CARIVGTHGFDYW	Poulsen.2007
TT	NA	CVSGSSLDYW	Poulsen.2011
TT	NA	CVSGGSLDYW	Poulsen.2011
TT	NA	CVRDYNGSGKYYFEYW	Poulsen.2011
TT	NA	CATGRTLDYW	Poulsen.2011
TT	NA	CATGNTLDYW	Poulsen.2011
TT	NA	CASGYCSSTSCYDYW	Poulsen.2011
TT	NA	CASGSTLDYW	Poulsen.2011
TT	NA	CASGRSLDYW	Poulsen.2011
TT	NA	CARWRWHQSEFDYW	Poulsen.2011
TT	NA	CARVYYDW	Poulsen.2011
TT	NA	CARTVRGVVPFDYW	Poulsen.2011
TT	NA	CARRYDFWSGFLDYW	Poulsen.2011
TT	NA	CARRHYCSSTSCYDAFDIW	Poulsen.2011
TT	NA	CARQTDNWFDPW	Poulsen.2011
TT	NA	CARIVGTHGFDYW	Poulsen.2011
TT	NA	CARIVGTHGFDYC	Poulsen.2011
TT	NA	CAREYGDYKFDYW	Poulsen.2011
TT	NA	CARDYYGSGSHYYFDYW	Poulsen.2011
TT	NA	CARDYSSPYYFDYW	Poulsen.2011
TT	NA	CARDYFGSGSVYYFDYW	Poulsen.2011
TT	NA	CARDYFGSGSIYYFDYW	Poulsen.2011
TT	NA	CARDHSSPYYFDYW	Poulsen.2011
TT	NA	CAKDLYGDYDLDYW	Poulsen.2011
Influenza	cross-reactive	CARGGRYYVDYFDYW	Corti.2010

Influenza	H2N2/H3N2	CARGISGSYGWFDPW	Krause.2012
Influenza	H1N1	CARHGYGDYVGYFDYW	Ohshima.2014
Influenza	H1N1	CARGPNYYESYFDYW	Ohshima.2014
Influenza	H1N1	CATSGTYYVSFFDYW	Ohshima.2014
Influenza	cross-reactive	CATGSGYYKRDYDYGMDVW	Pappas.2014
Influenza	cross-reactive	CASRYSSGWYYFDYW	Pappas.2014
Influenza	cross-reactive	CARILSADYYYGMDVW	Pappas.2014
Influenza	cross-reactive	CAKRYSSGWYSFDYW	Pappas.2014
Influenza	cross-reactive	CAGSGTYYVSRFDYW	Pappas.2014
Influenza	cross-reactive	CARGNYYYESSLDYW	Patent: US 8,192,927
Influenza	cross-reactive	CARGPHYYSSYMDVW	Patent: US 8,192,927
Influenza	cross-reactive	CARGPTYYYSYMDVW	Patent: US 8,192,927
Influenza	cross-reactive	CARGLYYYESSLDYW	Patent: US2011/0038935
Influenza	H1N1	CARHDSSGYHPLDYW	Thomson.2012
Influenza	H1N1	CARGPKYYHSYMDVW	Thomson.2012
